# Monoaminergic Modulation of Spinal Viscero-Sympathetic Function in the Neonatal Mouse Thoracic Spinal Cord

**DOI:** 10.1371/journal.pone.0047213

**Published:** 2012-11-05

**Authors:** Amanda L. Zimmerman, Michael Sawchuk, Shawn Hochman

**Affiliations:** 1 Department of Biomedical Engineering, Emory University/Georgia Institute of Technology, Atlanta, Georgia, United States of America; 2 Department of Physiology, Emory University, Atlanta, Georgia, United States of America; University of Cincinnatti, United States of America

## Abstract

Descending serotonergic, noradrenergic, and dopaminergic systems project diffusely to sensory, motor and autonomic spinal cord regions. Using neonatal mice, this study examined monoaminergic modulation of visceral sensory input and sympathetic preganglionic output. Whole-cell recordings from sympathetic preganglionic neurons (SPNs) in spinal cord slice demonstrated that serotonin, noradrenaline, and dopamine modulated SPN excitability. Serotonin depolarized all, while noradrenaline and dopamine depolarized most SPNs. Serotonin and noradrenaline also increased SPN current-evoked firing frequency, while both increases and decreases were seen with dopamine. In an *in vitro* thoracolumbar spinal cord/sympathetic chain preparation, stimulation of splanchnic nerve visceral afferents evoked reflexes and subthreshold population synaptic potentials in thoracic ventral roots that were dose-dependently depressed by the monoamines. Visceral afferent stimulation also evoked bicuculline-sensitive dorsal root potentials thought to reflect presynaptic inhibition via primary afferent depolarization. These dorsal root potentials were likewise dose-dependently depressed by the monoamines. Concomitant monoaminergic depression of population afferent synaptic transmission recorded as dorsal horn field potentials was also seen. Collectively, serotonin, norepinephrine and dopamine were shown to exert broad and comparable modulatory regulation of viscero-sympathetic function. The general facilitation of SPN efferent excitability with simultaneous depression of visceral afferent-evoked motor output suggests that descending monoaminergic systems reconfigure spinal cord autonomic function away from visceral sensory influence. Coincident monoaminergic reductions in dorsal horn responses support a multifaceted modulatory shift in the encoding of spinal visceral afferent activity. Similar monoamine-induced changes have been observed for somatic sensorimotor function, suggesting an integrative modulatory response on spinal autonomic and somatic function.

## Introduction

The central nervous system receives sensory information from the visceral organs through two paths: the vagus nerve, which projects to the nucleus of the solitary tract [1] and through sympathetic and pelvic parasympathetic nerves, which pass through prevertebral and/or paravertebral ganglia to the thoracolumbar and sacral spinal cord [2], [3]. It is commonly thought that nociceptive signals travel predominantly through the spinal cord path [4], and connect to somatic and sympathetic efferents through disynaptic and polysynaptic pathways [5], [6]. While the role of spinally projecting visceral afferents on nociception has received considerable attention [4], [7]–[9], little is understood on the role of visceral afferent pathways in modulating activity of primary afferents via presynaptic inhibition.

Thinly myelinated and unmyelinated visceral afferents [10], [11], [12] comprise a small percentage of dorsal root ganglia neurons in the thoracolumbar spinal regions [10], [11], [13], yet they project multi-segmentally and more diffusely than their somatic counterparts [14]–[16]. Visceral afferents have distinct spinal projection patterns and terminate in lamina I as well as in the deep dorsal horn (laminae IV–V), with a few collaterals reaching near lamina X [10], [15], [17]. The greater splanchnic nerve contains visceral afferents originating from the gut, pancreas, spleen, kidneys, testis/ovaries, and pelvic organs [8]. Stimulation of splanchnic nerve has been used to study visceral afferent inflow and has been shown to evoke both autonomic and somatic motor spinal reflexes [12], [18], [19], [20], [21].

Descending monoaminergic systems that release serotonin (5HT), noradrenaline (NA) and dopamine (DA) project densely to and have considerable modulatory actions on motor, autonomic, and sensory systems in both mammalian and non-mammalian species [22], [23], [24], [25]–[29]. Studies on visceromotor and pressor responses to colorectal distension in the awake rat have indicated antinociceptive actions of NA and 5HT [9], [30], and one study of spinal micturition reflexes has suggested inhibitory actions of DA [31]. The neuromodulatory role of these monoamines on intraspinal visceral afferents, interposed interneurons, and efferent activity needs to be specifically addressed to understand their integrative actions, yet there appear to be no systematic investigations on their site of action or dose-dependent modulation.

An effective means of inhibiting primary afferent influence on central circuits is via presynaptic inhibition of their intraspinal terminals. One form of presynaptic inhibition (PSI) is ionotropic, recorded as a summed back-propagated depolarization of primary afferent terminals termed primary afferent depolarization (PAD) [32]. PAD is traditionally thought to be mediated by last order GABAergic interneurons [32], [33], though ionotropic glutamate and 5HT_3_ receptors can also generate PAD [34], [35]. PAD has been shown in visceral afferents in response to splanchnic nerve and sympathetic chain stimulation [36]. Though descending monoaminergic systems have been shown to play a strong role on sensory processing in spinal interneurons [37], [38] and on modulating PAD in somatic afferents [39], [40], their modulatory actions on visceral afferent mediated PAD have not been determined.

We developed an *in vitro* spinal cord/sympathetic chain preparation in the neonatal mouse and stimulated visceral afferents in the splanchnic nerve or sympathetic chain to record evoked PAD from thoracic dorsal roots and reflex responses from thoracic ventral roots. Since activity in thoracic ventral root recordings indicate efferent population responses of both somatic and sympathetic efferents, we sought to further clarify monoamine actions specifically on sympathetic function by recording from sympathetic preganglionic neurons (SPNs).

SPNs integrate activity from descending and sensory systems to determine the final central output of the sympathetic nervous system [41]. SPNs are located predominantly in the thoracolumbar intermediolateral column in distinct clusters that form a ladder-like distribution symmetric around the central canal [42]. Descending dopaminergic, noradrenergic, and serotonergic systems have projections corresponding to the ladder-like distribution of SPNs, suggesting a direct neuromodulatory influence [43]–[46]. While direct and indirect actions have been reported for NA, 5HT and DA on SPNs, conclusions as to the overall actions are often contradictory, and may partly be attributed to age and species specific differences [47]–[53]. Here, we used an *in vitro* slice preparation recording from fluorescently- identified SPNs in a HB9-GFP transgenic mouse line as done previously [54] to correlate the effects of the monoamines on SPN excitability with visceral afferent-evoked actions from the same mice in the intact spinal cord.

## Materials and Methods

### Ethical Approval

All procedures described here comply with the principles of The Care and Use of Animals outlined by the American Physiological Society and was approved by the Emory University Institutional Animal Care and Use Committee.

### Splanchnic Nerve and Sympathetic Chain Immunohistochemistry

The splanchnic nerve and sympathetic chain connecting the rostral three ganglia were isolated using the dissection described below. Sympathetic chains were fixed in 4% paraformaldehyde for 1 hour, then cryoprotected in 10% sucrose plus 0.1 M PO_3_ (pH 7.4) and stored at 4°C. Before staining, chains were washed overnight in 0.1M PO_3_ buffered saline (PBS), then incubated with primary antibodies CGRP (goat, AbD Serotec, 1∶200), TH (rabbit, Millipore, 1∶1000), and anti-GFP (chicken, AbCam, 1∶1000) for 48 hours at 4°C, then washed three times in PBS containing 0.3% Triton X-100 for 30 minutes each at room temperature. Primary antibodies were stained with Alexa488 anti-chicken (diluted 1∶100), cy3 anti-goat (diluted 1∶250), and cy5 anti-rabbit (diluted 1∶100) secondary antibodies, all from Jackson Immunoresearch.

### In Vitro Spinal Cord and Sympathetic Chain Preparation

In order to examine net modulatory actions on thoracic spinal efferents (whose axons predominantly originate from SPNs) and visceral afferent evoked PAD, both ventral root efferent and dorsal root afferent responses to visceral sensory stimulation were assessed with DC recordings. Electrical stimulation of the greater splanchnic nerves has often been used to study visceral afferent inflow, and this paradigm was used again here.

#### Dissection

All experiments were performed at postnatal day (P) 5–8 mice crossed from transgenic hemizygote HB9-eGFP females (JAX laboratories) and inbred C57/BL6 males. Mice were either HB9-eGFP^+/−^ heterozygotes or wild type. Animals over age P6 were anesthetized with 10% urethane (2 mg/kg i.p.) before decapitation. All animals were decapitated and eviscerated, leaving only the vertebral column, ribcage, and surrounding tissues. The preparation was then placed in a perfusion chamber filled with low-calcium, high-magnesium artificial cerebrospinal fluid (aCSF) containing (in mM): 128 NaCl, 1.9 KCl, 1.2 KH_2_PO_4_, 26 NaHCO_3_, 0.85 CaCl_2_, 6.5 MgSO_4_, and 10 d-glucose (pH of 7.4). A dorsal laminectomy and ventral vertebrectomy were performed to expose the dorsal and ventral sides of the spinal cord, respectively, from the upper cervical region to the mid-sacral level. Care was taken to cut medial to the aorta on the left side, to preserve the connections of the dorsal and ventral roots to the sympathetic chain. The aorta was then carefully removed, and the surrounding fascia dissected away from the left sympathetic chain. The splanchnic nerve was identified branching laterally from the sympathetic chain at T_13_ and innervating the celiac ganglia, and cut midway between the sympathetic chain and the celiac ganglia. The perfusion solution was then switched to regular aCSF (128 NaCl, 1.9 KCl, 1.2 KH_2_PO_4_, 26 NaHCO_3_, 2.4 CaCl_2_, 1.3 MgSO_4_, and 10 d-glucose). Pancuronium bromide (25 µM) was added to limit movement of the preparation.

#### Recording Configuration

**Figure 1:**
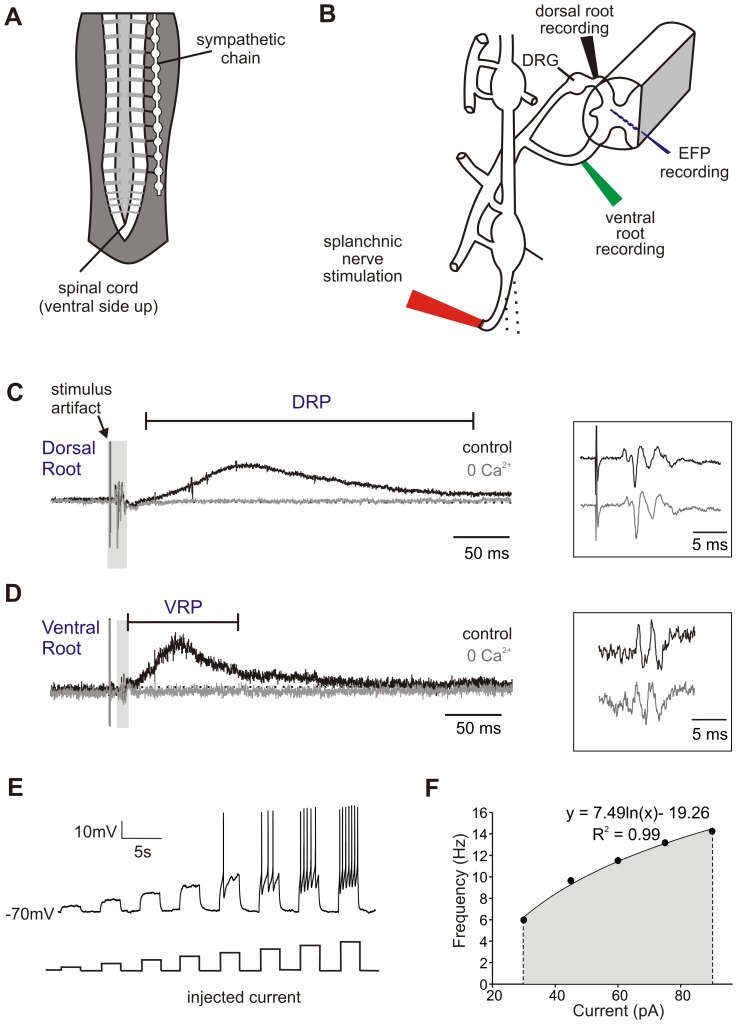
Sympathetic chain anatomy and example recordings. A. Schematic of the sympathetic chain and its connections to the spinal cord. B. Recording configuration, with suction stimulating electrode on greater splanchnic nerve and suction recording electrodes on dorsal and ventral roots. Field potential electrode penetrates into dorsal horn from cut surface of spinal cord. C and D. Sample recorded dorsal root potential (DRP) recorded at dorsal root entry zone (C) and ventral root potential (VRP) recorded at ventral root near exit from cord (D), both of which disappear in nominally 0 Ca^2+^ aCSF (grey traces). Higher magnification of grey highlighted orthodromic afferent (C) and antidromic efferent (D) spiking is shown in boxes at right. E. Whole-cell recorded response of a single SPN in spinal slice to increasingly depolarizing current steps (multiples of 5 pA, 1 s duration). Bottom trace shows current steps. F. Mean firing frequency for each current step plotted. Data points are best fit with a logarithmic line (equation shown). Area under this line (shaded in grey) is calculated by integrating line from first to last data point.

The recording configuration is shown in Figure 1B. Bipolar glass suction electrodes (inner diameter 60–100 µm) were used to both record slow potentials in spinal roots and stimulate the splanchnic nerve or sympathetic chain. Teflon insulated and chlorided silver ground wires were wrapped around the outside of the glass to reduce fluctuations in the DC recordings and minimize current spread to intercostal muscles. Visceral afferents were activated by stimulating the splanchnic nerve or other cut regions of the sympathetic chain at a rate of .0167 Hz (once every 60s), as responses to higher frequency stimuli were susceptible to depression [55]. Responses were recorded from the T_11_–T_13_ dorsal and ventral roots.

Due to the mixed afferent/efferent composition, electrical stimulation of the splanchnic nerve and caudal sympathetic chain evoked short-latency orthodromically and antidromically propagating population spikes in dorsal root and ventral root recordings, respectively (Figure 1C and D). Direct electrical recruitment of axons was verified by their persistence following block of chemical synaptic transmission after exchanging the bath to a nominally Ca^2+^-free aCSF. Orthodromic afferent volleys from splanchnic nerve were recorded in dorsal roots as far rostral as T_6_, confirming direct afferent axonal projections to multiple spinal segments. Recorded DRPs from dorsal roots are produced as a result of electrotonically back-propagating depolarizing potentials (PAD) from intraspinal primary afferent terminals. PAD leads to presynaptic inhibition by reducing transmitter release [32], so the slow DRPs were used as a measure of the magnitude of PAD-evoked presynaptic inhibition. DRPs were recorded with a suction electrode attached *en passant* to the root as close to the entry zone as possible to minimize the distance of the recorded electrotonically decaying potential. Similarly recorded ventral root potentials (VRPs) were interpreted as compound excitatory postsynaptic potentials (EPSPs) of the combined captured axonal population of somatic and sympathetic efferents. Examples of recorded DRPs and VRPs are shown in Figure 1C and D, respectively.

Neural activity was collected on a custom built 4 channel direct current amplifiers, low pass filtered at 3 kHz, and digitized at 5 kHz (Digidata 1440) and recorded in Clampex for off-line analysis (Molecular Devices).

#### Extracellular Field Potentials

When extracellular field potentials (EFPs) were recorded, a 2/3 sagittal section of the spinal cord was completed using fine insect pins. Micropipettes (tip diameter 1–2 µm, resistance 4–7 MΩ) were filled with 2 M KCl and penetrated the cut surface of the spinal cord at an approximately 35° angle until EFPs were seen in response to splanchnic nerve or caudal sympathetic chain stimulation. EFPs reflect population membrane voltage changes in the neurons around the tip of the electrode. Recording locations were approximated after the experiment using a transverse picture of the sectioned spinal cord, distance from the surface of the spinal cord marked during the experiment, and angle of micropipette penetration (see Figure 1B).

#### Drug Solutions and Applications

Stock solutions of drugs (10–100 mM) were made and stored at −20°C until needed. All drugs were dissolved in regular ACSF and perfused through the gravity perfusion line. For time response trials, 5–10 µM of 5-hydroxytryptamine HCl (5HT), norepinephrine bitartrate (NA), or dopamine HCL (DA) were applied for 10 minutes, then washed out with regular ACSF that continued to contain 25 µM pancuronium for at least 30 minutes. For dose-response trials, increasing dosages of 5HT, NA, or DA were applied cumulatively, with 10 minutes in between dose increments. In a few experiments, bicuculline (10–20 µM) was added to block GABA_A_ receptor mediated transmission. All drugs were purchased from Sigma Aldrich.

#### Data Analysis

Changes were assessed in the evoked DRPs, VRPs, and EFPs using the following paradigm. A custom built MATLAB program was used to subtract the baseline values prior to the stimulus, low-pass filter the response at 100 Hz, find the onset of the evoked slow potential, their peak response, and their integral under the filtered response from onset to offset (defined as the time at which the slow potential decayed to 1/3 peak amplitude). This program and cutoff frequency was found to accurately capture these slow potentials. Responses were averaged for the last 5 minutes of each drug dose increment for dose response curves. For time-dependent analysis, responses were averaged over five minute intervals. In order to minimize effects of differences in suction in the dorsal and ventral root recording electrodes, and differences in EFP recordings, all evoked potentials were normalized to baseline values. Statistics were completed using a two-tailed paired t-test in Microsoft Excel. Timing of 1^st^ afferent spikes was assessed visually in Clampfit (Molecular Devices).

### Slice Electrophysiology

#### Dissection

All experiments were performed in transgenic mice expressing HB9-eGFP (JAX laboratories; known to label SPNs), postnatal day 3–9 as described previously [54]. Briefly, neonatal animals were decapitated, eviscerated, and the spinal cords removed, and a T_8_-L_2_ section isolated and sliced into thick transverse sections (400 µm). Initial removal of the spinal cord and slicing were performed in cooled, oxygenated sucrose aCSF, containing (in mM): 250 sucrose, 2.5 KCl, 2 CaCl_2_, 1 MgCl_2_, 25 glucose, 1.25 NaH_2_PO_4_, and 26 NaHCO_3_, pH 7.4. Slices were left to recover for at least 1 hour.

**Figure 2:**
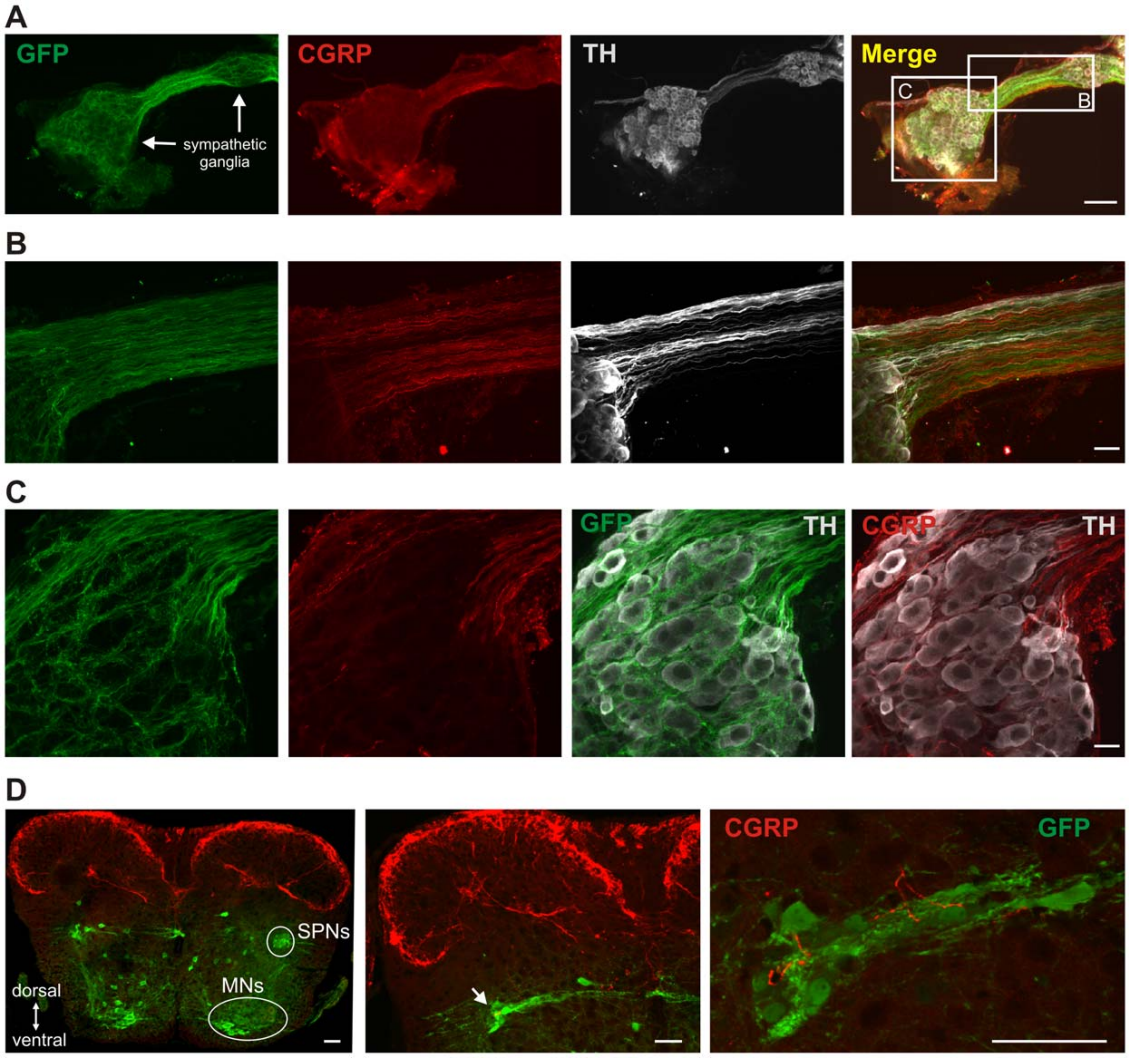
Axon fiber composition in paravertebral ganglia and related labeling in spinal cord. A. Lower power image of two caudal sympathetic ganglia and connecting nerve bridge showing nerve contains a mixture of at least 3 neurochemically distinct fiber populations: HB9-GFP+ sympathetic preganglionic neurons (SPNs), CGRP+ visceral afferents, and TH+ sympathetic postganglionics. Boxed regions are enlarged in B and C. B. Enlargement showing SPNs (GFP+), CGRP and TH immunolabeled axons in axon bundle between two ganglia. Image represents composite of 73 consecutive confocal images taken at 0.38 µm optical section thickness (27.74 µm total thickness). C. Enlargement of SPNs (GFP+), CGRP and TH immunolabeling in the left sympathetic ganglion. Section in central region of ganglia shows that, while SPNs appear to form basket-like synapses around postganglionics, CGRP+ afferents do not project through this region. Image is a collapsed stack of 11 consecutive confocal images (4.18 µm total). D. CGRP (red) and HB9-GFP labeling (green) in a transverse section of thoracic spinal cord. Note predominant CGRP labeling in the dorsal horn. HB9-GFP labeling of SPNs in the intermediolateral nucleus and motoneurons (MNs) in the ventral horn are identified on the right side. Middle panel shows CGRP labeling extending ventrally including into the intermediolateral nucleus (at arrow) while right panel provides a higher magnification image showing CGRP labeling intermingled with SPNs in the intermediolateral nucleus as well as along its medially oriented SPN projections. Scale bar is 100 µm in A, 20 µm in B and C, and 50 µm in D.

The recording chamber was continuously perfused with oxygenated aCSF (composition specified above) at a rate of ∼2 ml/minute. Patch clamp recordings were made from fluorescently-identified SPNs located within the intermediolateral nucleus (e.g. Fig. 2D). The intracellular recording solution was comprised of (in mM): 140 K- gluconate, 11 EGTA, 10 HEPES, 1 CaCl_2_, and 35 KOH, pH 7.3. Cells were brought to −70 mV holding potential by injecting bias current and recorded in gap-free mode to assess effects on membrane potential. Intracellular current was injected in 1 s rectangular waveforms and incremented in magnitude in a stepwise fashion (5–30 pA increments, depending on input resistance [54]). Changes in membrane properties and excitability were quantified. Using current step sequences applied before and during drug application, mean firing frequency was calculated for each current step. Frequency – current (*f*-I) plots were then fit with a logarithmic trendline using Microsoft Excel. Matlab was then used to integrate the area under the logarithmic trendline both before and during drug application (Figure 1E, F). This integrated area was then used to quantify changes in cellular excitability, with statistical significance found using a students' paired t-test.

#### Application of Agonists

5HT, NA, and DA were bath applied at 10 µM, a concentration believed to be below the concentration where nonspecific binding actions have been observed [37], [56]. Each agonist was applied for 1–3 minutes, and a washout period of 10–20 minutes was allowed between drug applications. Drug order was random, and often only one or two agonists were used per cell, due to the time constraints of the recordings.

## Results

### CGRP^+^ visceral afferents travel through sympathetic ganglia

While many studies have shown that visceral afferents project to dorsal root ganglia (DRG) several segments away from their spinal nerves [14]–[16], few have suggested they reach the appropriate DRGs by traveling within the sympathetic chain [2], [57]. We therefore sought to confirm the presence of afferents in the sympathetic chain and major splanchnic nerve in the neonatal mouse model system, using immunohistochemistry on fixed tissue of dissected whole mounts of the major splanchnic nerve and sympathetic chain.

Calcitonin gene-related peptide (**CGRP**) is a peptide found in about 40–50% of DRG neurons, with a strong preference for labeling visceral afferents [58], [59]. CGRP^+^ staining was compared to tyrosine hydroxylase (**TH**), a marker for sympathetic postganglionic neurons [60], and to HB9-GFP, which labels sympathetic preganglionics [61]. Triple labeling results showed that axon bundles between ganglia contained considerable numbers of all three axon fiber types (Figure 2A–C). Within the ganglia, while many preganglionic GFP^+^ axons avoided the TH^+^ somas entirely, many also appeared to surround and likely synapse on them. In contrast, almost all CGRP^+^ axons projected outside the region of TH^+^ postganglionic somas, and there was no evidence of CGRP^+^ afferents forming synapses on postganglionic cell somas (Figure 2). Additionally no overlap in labeling was seen for any of these makers, cleanly identifying these 3 axon fibers as distinct neurochemically-identifiable populations. Most importantly, our immunohistochemistry clearly demonstrates that CGRP^+^ afferents projecting from the splanchnic nerve enter the CNS through the spinal cord via the sympathetic chain, thereby allowing for stimulation of the sympathetic chain and greater splanchnic nerve for selective stimulation of visceral afferents.

The distribution of CGRP labeling in the thoracic cord is shown in a transverse section. Note the dense labeling in the superficial dorsal horn, with additional labeling in the deep dorsal horn, and in lamina X near the central canal (Figure 2D). Note also the presence of sparse CGRP+ axons in the intermediolateral nucleus where the majority of SPNs are found. Splanchnic CGRP+ afferent labeling is not associated with superficial dorsal horn labeling [62].

### Monoamines depress ventral root potentials

**Figure 3:**
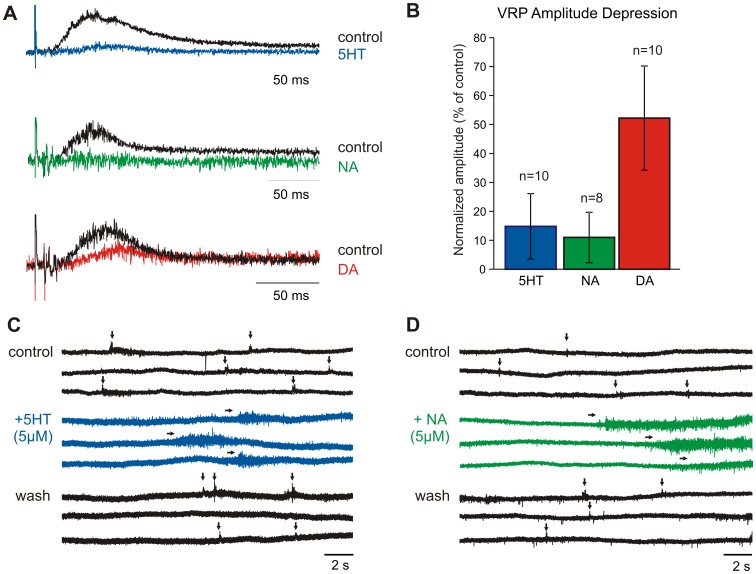
Monoaminergic modulation of ventral root activity. A. Example visceral afferent-evoked responses recorded from T12 ventral roots, and their modulation by 5HT, NA, and DA. Control is black, drug is in blue, green, or red, respectively. B. Combined evoked depression with 10 µM 5HT, NA, and DA across all time and dose-response trials, with standard deviation bars and number of trials displayed. C. Example change in spontaneous ventral root activity after addition of 5HT. Shown are 3 consecutive epochs during 5 minute periods for control, after 5HT (5 µM) was applied, and 15 minutes after washout. Horizontal arrows denote emergent motor bursts observed in the presence of 5HT. Vertical arrows denote spontaneous ventral root potentials, some of which reach spike threshold. D. Example change in spontaneous ventral root activity after addition of NA. Note that both 5HT and NA increased background spiking and the emergence of bursting events, while eliminating spontaneous potentials. DA had limited effects on spontaneous activity and is not displayed.

Application of 5–10 µM 5HT, NA, and DA significantly depressed visceral afferent- evoked VRPs recorded from T11, T12 or T13 spinal segments (Figure 3A). While the rostrocaudal extent of evoked responses was not systematically studied, evoked VRPs were seen bilaterally, and as rostral as T3 and caudal as segment S4. Combining all dose-response and time-response trials, VRP amplitude was reduced to 14.8±11.3% of control values for 5HT, 11.0±8.7% for NA, and 52.2±18% for DA. Interestingly, 5HT and NA also greatly depressed ongoing spontaneous VRPs while concomitantly increasing spontaneous spiking activity in the ventral roots (n = 4/4 and 3/3, respectively) (Figure 3C,D), overall supporting the notion that 5HT and NA have excitatory actions on efferent neurons (both somatic and autonomic), while depressing visceral afferent evoked reflexes.

### Monoaminergic Actions on Spinal Sympathetic Efferents

In both the rodent and human thoracic spinal cord, the number of SPN axons in the ventral roots greatly exceeds those of somatic motoneurons [63], [64]. Still, recordings from ventral roots make it impossible to determine whether the monoaminergic actions are on sympathetic preganglionic neurons, motor neurons, or some combination of both. Given the species-specific and seemingly contradictory actions of the monoamines previously reported on SPNs [47], [48], [52], we sought to characterize the effects of 5HT, NA, and DA on SPN intrinsic properties in the neonatal mouse and more clearly elucidate the effects of the monoamines on sympathetic output in the HB9-GFP mouse model. Accordingly, SPNs in the intermediolateral column were targeted for whole-cell patch clamp recordings in transverse slices of thoracic spinal cord as reported previously [54].

#### Serotonin

**Figure 4:**
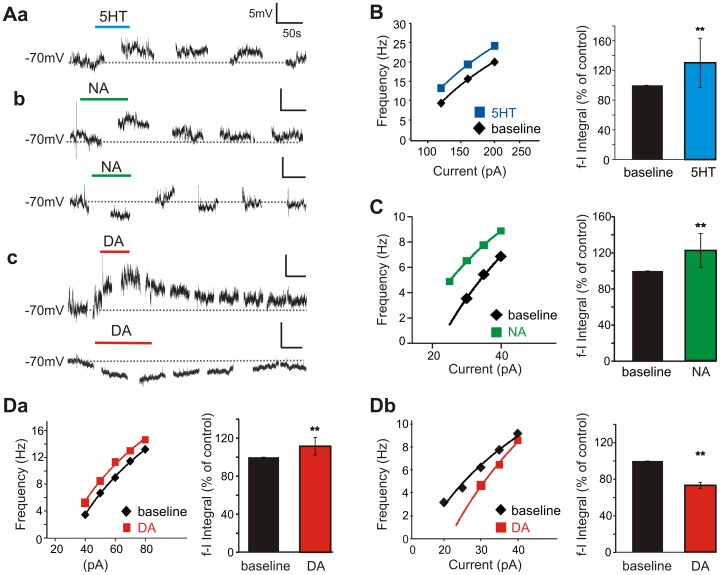
Monoaminergic modulation of SPNs. A. Sample of voltage traces recorded when 5HT, NA, or DA (10 µM, 60–90s) was added to the bath. Interruptions in trace represent periods where current step protocols were performed. Aa. 5HT depolarized the cell membrane in all cells tested. Ab. NA primarily depolarized the membrane, but hyperpolarizations were also seen. Ac. DA both depolarized and hyperpolarized membrane potential. B. 5HT increased cellular excitability. Shown is mean firing frequency in response to series of current steps (5–30 pA steps,1s duration) in control and during peak of 5HT application. Line demarks best fit logarithmic line, and the area under this line was calculated by integrating from first to last data point. Plot on right shows normalized integral changes with standard deviations. C. Mean firing frequency changes in the presence of NA. Regardless of action on membrane potential, NA shifted the f-I plot up and to the left. D. While DA increased cellular excitability in the majority of neurons (a), decreased excitability was also seen (b). ** denotes p<0.05 from a paired t-test applied to control and monoaminergic induced areas.

With the membrane potential held at −70 mV, bath application of 10 µM 5HT depolarized all SPNs tested (mean 4.9±2.1 mV, n = 6; Figure 4Aa). Compared to baseline values, the frequency-current (*f*-I) plots were also shifted up to the left, i.e. SPNs fired action potentials at a lower current injections and at higher rates. 5HT increased the firing response to current injection by a mean 15.9±9.2% (p = 0.04, n = 6; Figure 4B).

#### Noradrenaline

Bath application of NA had more diverse actions on SPN excitability. NA primarily depolarized the membrane (mean 4.7±2.2 mV, n = 4), yet hyperpolarizations (mean 3.1±0.1 mV) were also seen (n = 2) (Figure 4Ab). Regardless of the effects on the membrane, NA shifted the cell's *f*-I plot up in all neurons tested, by a mean 22±20% (p = 0.008, n = 6). This can be seen in Figure 4C.

#### Dopamine

DA bath application lead to both depolarizations (mean 6.0±3.5 mV, n = 7) and hyperpolarizations (mean 5.2±0.9 mV, n = 2) in SPNs (Figure 4Ac). In contrast to 5HT and NA, while DA increased the cell's firing frequency in response to current injection in the majority of neurons (mean 11.5±9.2%; p = 0.004, n = 8), decreased responses were also seen (mean −27.2±3.4%; p = 0.05, n = 3), as shown in Figure 4D. Changes in excitability were not correlated to changes in membrane potential.

Overall, 5HT, NA, and usually DA, significantly increased SPN firing in response to current injection. These upward-shifted *f*-I plots would support a monoamine-induced increased sympathetic firing to a given excitatory drive.

### Dorsal Root Potentials and Primary Afferent Depolarization

Given that the monoamines predominantly increased sympathetic efferent (SPN) excitability but decreased visceral afferent-evoked efferent synaptic responses (↓VRP), we assessed the actions of the monoamines on visceral afferent transmission and PAD-mediated presynaptic inhibition. Returning to the spinal cord and sympathetic chain preparation, we measured DRPs in the dorsal root as a measure of PAD.

#### Initial Characterization and Comparison to VRP

**Figure 5:**
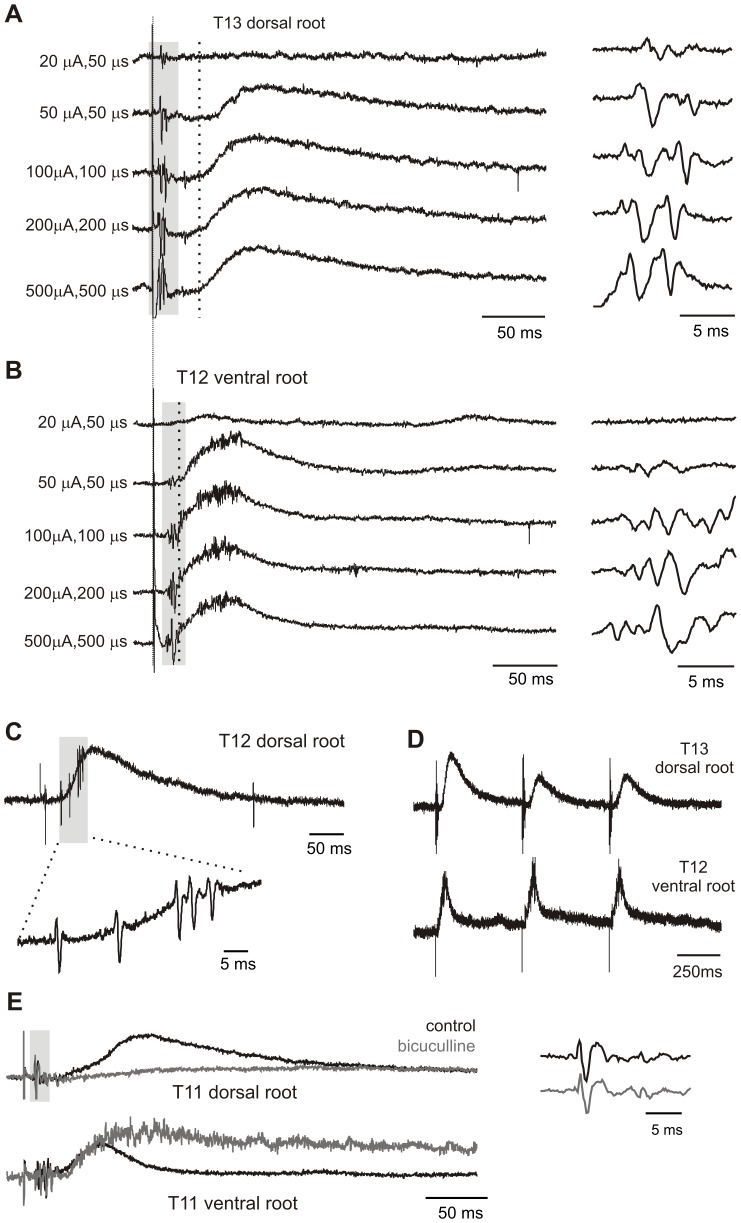
Differences in properties of DRPs and VRPs. A. Increasing stimulus intensities lead to increased afferent volleys and subsequent DRPs. Each trace is an average of 5 events. Note intensities greater than 100 µA, 100 µs increase afferent volley amplitudes (grey shaded region), but not the DRP. Note also that top trace has an afferent volley but no DRP. B. Increasing stimulus intensities and effects on the VRP in the same animal. Notice onset and duration difference between DRP and VRP (dotted vertical lines in A and B). Grey shading denotes orthodromic volleys, seen at higher magnification on the right. C. Single trace of an evoked dorsal root response from another experiment. Grey box and higher magnification below highlight observed dorsal root reflexes associated with the DRP. D. The DRP undergoes frequency dependent depression (here with 2 Hz stimulation). Note this is not the case for the VRP, which can follow frequencies up to 5 Hz. E. The DRP but not VRP is blocked by bicuculline. In example shown, each trace is an average of 10 sweeps, for control and 5 minutes after 20 µM bicuculline. Closeup of afferent volley at right highlights that bicuculline has no effect on the afferent recruitment.

Visceral afferent stimulation-evoked DRPs were widespread throughout the thoracic spinal cord, as DRPs were recorded at the spinal levels sampled (T_9_–T_13_) with similar shape and onset. This suggests that visceral afferent stimulation-induced PAD has a broad distribution, and is consistent with the reported actions of somatosensory evoked PAD [65]. While afferent volleys could be recruited at stimulus intensities as low as 8 µA, 50 µs, greater stimulus intensities were often required to elicit a DRP (Fig. 5A). Figure 5 provides an example of the relationship between stimulus intensity and the recruitment of spike volleys, DRPs, and VRPs. Stimulus intensities above 100 µA/100 µs did not further increase DRP recruitment (Figure 5A), so this value was chosen for subsequent studies on neuromodulation. On average, the DRP onset was 31.7±6.1 ms after arrival of the first afferent volley, while the VRP onset was at 11.4±8.0 ms (n = 17). The onset of the VRP preceded the DRP therefore by 19.8±8.4 ms, suggesting that the circuitry responsible for evoking the VRP includes a distinct shorter latency pathway. DRPs lasted on average 565.6±11.8 ms, reaching their peak values 50.1±10.1 ms after onset. The evoked DRP was accompanied by dorsal root reflexes in 11/18 cases, (Figure 5C). Dorsal root reflexes commonly represent an intraspinal primary afferent depolarization of sufficient magnitude to be supra-threshold for antidromic action potential initiation in afferent terminals.

In addition to being quicker in onset than the DRP, the VRP had a lower threshold for recruitment in all stimulus intensity trials (n = 4/4). Also, while the visceral afferent-evoked VRP could follow stimulation up to 5 Hz without depression, the DRP was unable to follow stimulus frequencies greater than .0167 Hz (see Figure 5D). Overall, these differences in onset, frequency sensitivity and threshold for recruitment all support the notion that evoked DRPs and VRPs are mediated at least partly by distinct circuits.

#### GABA_A_ Sensitivity

To test whether PAD induced by visceral afferents is GABA_A_ receptor mediated, 10–20 µM of the competitive antagonist bicuculline was added. In all cases, bicuculline greatly depressed the DRP (mean peak depression to 20.3±3% of control values, n = 4/4). Bicuculline evoked actions were mediated by spinal circuits as there was no change in the recruitment or appearance of recorded afferent volleys. As expected, disinhibition simultaneously greatly facilitated evoked ventral root activity (Figure 5E).

#### Extracellular Field Potentials

**Figure 6:**
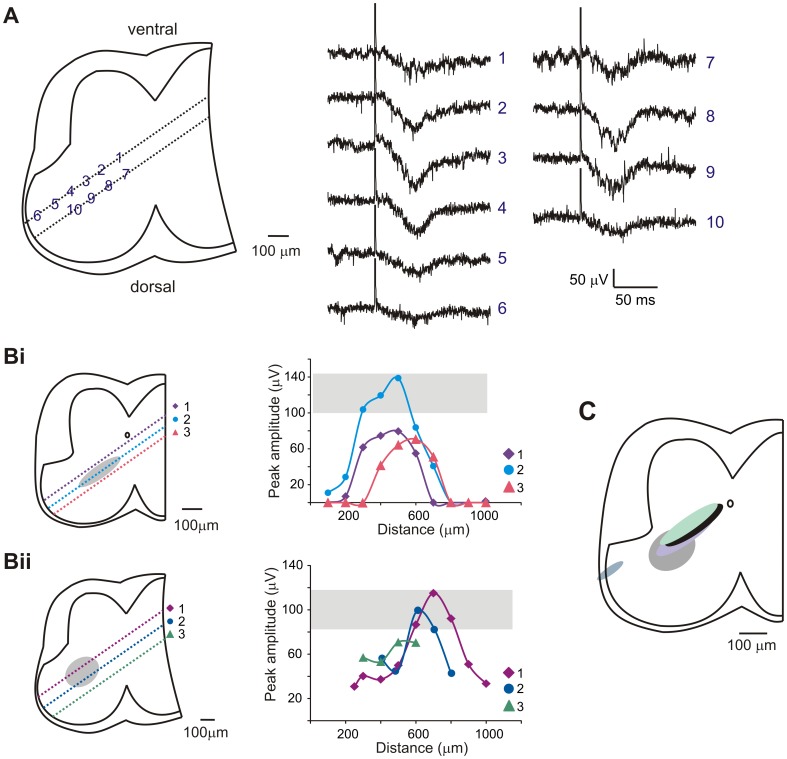
EFP recording locations. A. Sample traces of EFPs evoked at various recording locations marked by numbers. Each trace is an average of 5 events. B. Peak amplitude of evoked EFPs for 2 separate experimental days. Picture on left shows microelectrode paths denoted by separate numbers. Plot on right displays peak amplitude of EFP versus distance into the cord the microelectrode traveled. Grey box and oval denote largest EFP amplitudes and their locations recorded. Bii corresponds to same experiment as traces in A. C. Composite sketch estimation of where maximal EFPs were recorded, compilation of 5 experiments.

Intraspinal electrodes were positioned to examine monoamine transmitter modulation of population synaptic transmission, recorded extracellularly as EFPs. Short-latency EFPs have been used to report population monosynaptic afferent transmission [66]–[69]. The electro-anatomic location of splanchnic visceral afferent-evoked EFPs was estimated using systematic field potential tracking; with starting locations 100–400 µm below the ventral surface of the spinal cord in dorsally-angled penetrations up to depths of 1100 µm. Sample recordings from two tracks in the same preparation can be seen in Figure 6A. Figure 6B shows approximate regions of the spinal cord where EFPs were recorded and their relative peak amplitudes in two separate preparations. EFP amplitudes were consistently estimated to be maximal in the deep dorsal horn, with evidence of an earlier arriving EFP in some tracks in the superficial dorsal horn (Figure 6C). This is consistent with known afferent termination sites of visceral afferents including CGRP+ afferents (Figure 2D) [10], [15], [17], [62]. The earliest onset EFP in the deep dorsal horn was found to occur 4.5 ms after the first dorsal root afferent volley was seen, with a mean of 11.0±6.6 ms across trials. The largest amplitude EFPs were not necessarily the earliest in onset, with a mean onset of 12.9±5.8 ms after the first afferent spike was seen. Responses occurred on average 20.8±8.8 ms before onset of the DRP and often began before the VRP. Synaptic transmission at room temperature requires ∼3 ms [70], [71], so the observed central latency is sufficiently long enough to include oligosynaptic actions but may also reflect monosynaptic actions from slower conducting afferents. Regardless of whether the EFP reflects exclusively monosynaptic actions or not, their modulation would indicate actions on synaptic transmission in deep dorsal horn neurons.

### Monoaminergic Depression of Evoked Responses

**Figure 7:**
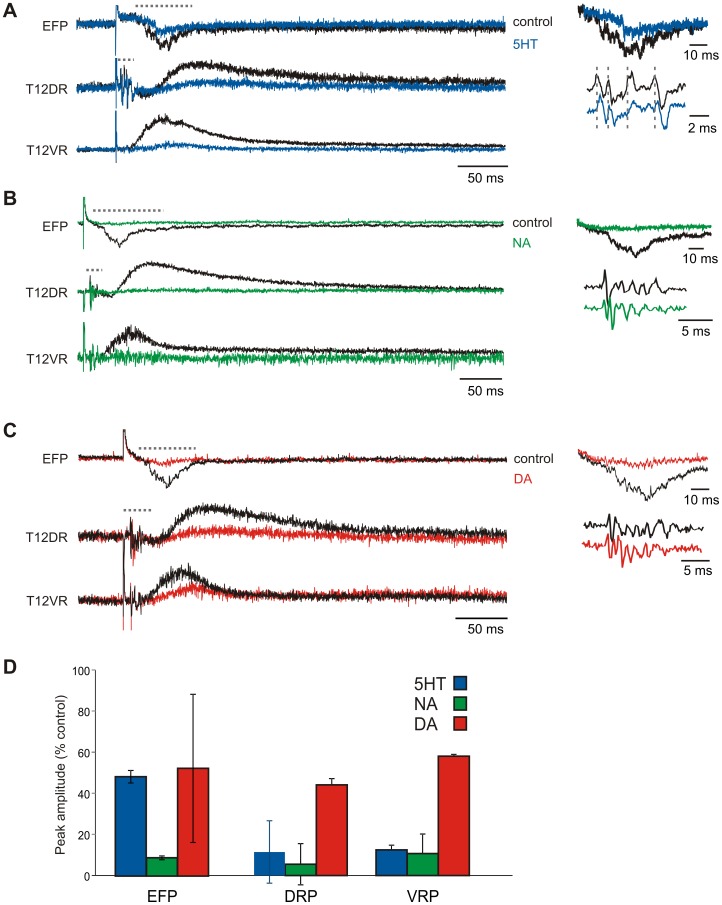
Monoamine effects on splanchnic evoked responses. A. Example of 5HT application on EFP, DRP, and VRP, control traces in black. Horizontal dotted lines identify magnified areas on the right for EFP (top) and DR afferent volleys (bottom). Note slight delay in afferent volley timing in the presence of 5HT. B. Example of NA application. C. Example of DA application. D. Comparison of effects of the monoamines on depression of EFP, DRP and VRP. Values are reported as mean with standard deviation bars.

Overall, all visceral-afferent evoked responses were depressed by the monoamines (Figure 7). As these actions were not accompanied by a change in afferent volley amplitudes, modulatory actions were via central mechanisms. The VRP depression by all three monoamines was also accompanied by actions on the visceral afferent evoked DRP and EFP. At 5–10 µM, both 5HT and NA nearly fully blocked the DRPs (to 12.7±13.4%, n = 5, p = .04 and 5.5±2.3%, n = 4, p = 0.03, of control values, respectively). DA depressed the DRP amplitude but to a much lesser degree (to 44.1±24.9% of control; n = 7, p = .01). NA also greatly depressed the EFP (8.8±0.9% of control, p = 0.03), comparable to its depression of the DRP. In contrast, EFP depression by 5-HT was much less (48.3±3.0%; p = 0.02) than its depression of the DRP. Thus, the reduction in DRP by NA associates strongly with depressed afferent transmission in the deep dorsal horn while additional projection territories or downstream sites also contribute to 5-HT's actions.

DA had variable effects on the EFP: substantial depression in 2/5 preparations, partial depression in 2/5 preparations and facilitation in 1/5 preparations, resulting in a mean depression of 52.3±36.0% (n = 5; p = .03; Figure 7D).

Given the common presence of multiple EFPs having different latencies of onset, we questioned whether the EFP depression calculated based on changes in overall peak amplitude was representative of all evoked EFP populations. Filtered and averaged EFP traces were therefore integrated in 5 ms bins, and depression was compared across bins. No statistically significant changes were seen, indicating a uniform depression of all temporal components of the EFP (data not shown).

**Figure 8:**
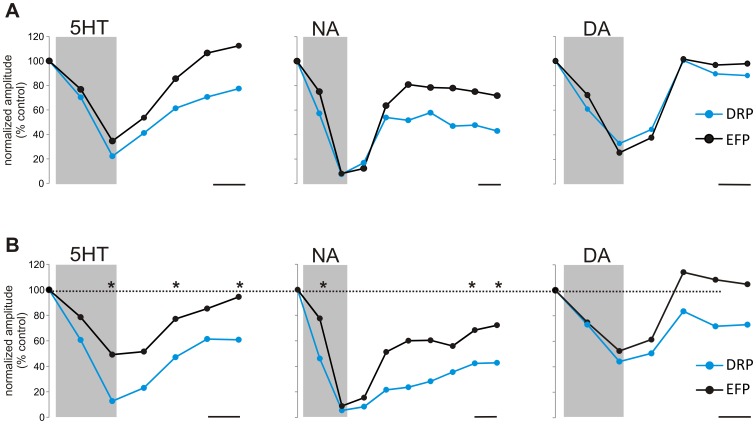
Comparing time and magnitude differences in monoamine-induced depression of DRPs and EFPs. Data points were obtained for each trial by averaging 5 stimulation episodes (1 minute per episode) and plotting the normalized peak amplitude. Monoamines were applied during the shaded periods. A. Time dependent depression of DRPs (blue) and EFPs (black) in an individual animal for 5HT, NA, and DA. B. Average data across animals (n = 4, 3, 4, respectively). * indicates p<0.05 when paired t-test was completed for each time point. No differences in time course were found for DA. Scale bars are 5 minutes in all panels.

A time-dependent comparison was made of the relative monoamine-induced DRP and EFP depression in the same animals (Figure 8A), with data binned at 5 minute intervals. For 5HT, the peak depression of the DRP was greater and time of depression more maintained compared to the EFP (Figure 8B). In comparison, while NA depressed both the DRP and EFP to a similar extent, the DRP was depressed more rapidly, and like 5HT, the depression was maintained for a longer time than that of the EFP (Figure 8B). No statistically significant changes were seen in the time course of DA-induced depression of the DRP and EFP. Collectively, the differentiable temporal and magnitude differences in the depression of the DRP compared to the EFP for 5HT and NA support partially independent sites of actions (see Discussion).

Similar to actions reported for 5HT in lumbar spinal cord in the neonatal rat [72], both 5HT and NA depressed spontaneous DRPs in all cases where spontaneous DRPs were evident (n = 3/3 for each; not shown). Whether these DRPs were initiated by ectopic firing in primary afferents and/or exclusively via intrinsic spinal circuits was not investigated.

### Dose Response Relations

**Figure 9:**
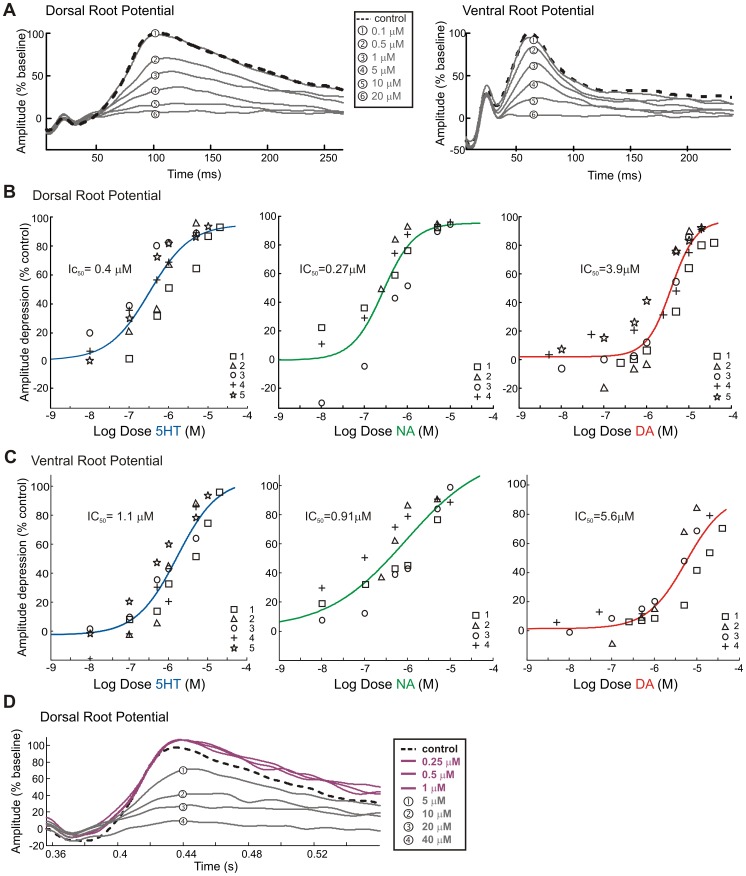
Dose-dependent actions on splanchnic evoked dorsal and ventral root potentials. A. Sample of dorsal root potentials (left) and ventral root potentials (right) evoked by splanchnic nerve stimulation. Each trace is the mean, normalized, and filtered trace of 5 sweeps for each dose increment, after 5 minutes of drug application. Control trace is dashed and other traces are number coded to corresponding dosage. B &C. Dose response curves for 5HT, NA, and DA, with each trial a distinct marker. Amplitude depression is shown as a % of control values for dorsal root (B) and ventral root (C) peak values. Line is a best fit dose-response equation to all data points, IC_50_ value is the dose at which the evoked response is half the control value. Note 10 fold increase in IC_50_ values between NA and DA for DRP depression. D. Sample dorsal root potentials for DA application. Control trace is dashed. Magenta traces denote low doses of DA which facilitated the DRP. Facilitation at low doses was seen in 3/5 trials. Numbers are coded to corresponding higher doses.

To determine relative efficacies of DRP and VRP depression, cumulative dose-response curves were generated for 5HT, NA, and DA. Figure 9A shows a sample of a cumulative dose-response trial for 5HT in a single preparation. When the evoked response was normalized to the initial slow potential amplitude and compared across trials, mean IC_50_ values of 0.33 µM and 1.7 µM were calculated for 5HT on the DRP and VRP, respectively. NA had similar dose-response relations with IC_50_ values of 0.27 µM and 0.91 µM, respectively (n = 4) (Figure 9B and C). In comparison to 5HT and NA, much higher concentrations of DA were needed to suppress the evoked responses. Moreover DA had a biphasic action on the visceral afferent-evoked DRP. At low concentrations (i.e. <5 µM) DA had little effect if any, and even facilitated the DRP in the majority of trials (n = 3/5; see highlighted region in Figure 9D). On the other hand, at higher concentrations, DA dose-dependently depressed the DRP. The best-fit dose response curve calculated an IC_50_ value of 3.9 µM.

## Discussion

Spinal visceral afferents encode physiologically relevant events in the viscera and transmit this to the CNS, leading to organ regulation (including via spinal reflexes) and occasionally conscious sensation [1]. Yet, except for sacral afferents, most spinal visceral afferents are thought to lack functional specificity, responding to mechanical, chemical, and sometimes thermal stimuli (e.g. [73], see [60] for review). While some visceral afferents are active under resting conditions, many also relay visceral pain information [60], [74], indicating that central mechanisms must exist to differentiate between organ regulation, non-painful sensation, and visceral pain. Indeed, organ specific presynaptic inhibition of afferents has been implicated in the control of micturition [75], [76], and mutual inhibition has been demonstrated between visceral and cutaneous afferents, presumably via presynaptic mechanisms [36].

To explore the neuromodulatory control of visceral afferent encoding, this study used a novel *in vitro* spinal cord with intact sympathetic chain preparation in the neonatal mouse. An *in vitro* approach allows for greater accessibility and pharmacological control of the environment as well as the avoidance of anesthetics that alter the GABA_A_ receptors involved in PAD generation [77], [78]. Using this system, we characterized spinal visceral afferent stimulation-evoked ventral root responses (reflexes and subthreshold VRPs), synaptic transmission in the dorsal horn (EFPs), as well as PAD-mediated presynaptic inhibition (DRPs). To our knowledge, we also undertook the first studies on modulation of visceral afferent-evoked PAD by the monoamine transmitters 5HT, NA, and DA. Overall, we observed that all three monoamines acted to suppress visceral afferent-evoked responses while concomitantly increasing ventral root spiking and SPN excitability. 5HT, NA, and DA were also found to inhibit visceral afferent evoked synaptic transmission in the dorsal horn and PAD. In sum, 5HT, NA and DA had similar actions and modulated several spinal pathways to provide a global modulatory shift in the autonomic regulation of visceral afferent and sympathetic efferent function.

Triple labeling for CGRP, TH, and GFP in HB9-GFP transgenic mice indicated the presence of 3 distinct fiber populations that travel between the paravertebral sympathetic ganglia. CGRP^+^ visceral afferents did not appear to synapse on TH^+^ sympathetic postganglionic neurons, often avoiding postganglionic cell bodies entirely, unlike direct synapses reported between afferents and postganglionic neurons in pre-vertebral ganglia [79], [80]. Consistent with the presence of CGRP^+^ afferents, splanchnic nerve stimulation induced afferent volleys, DRPs, and VRPs (often with superimposed reflexes) in multiple spinal segments, supporting known projections of afferents originating in the greater splanchnic nerve [2], and demonstrating that splanchnic afferents act over their broad projection territory. The rostral and caudal extent of these actions remains to be characterized systematically in mouse, but in cat it is generally from T3-L1 dorsal roots [2]. Given the relative scarcity of visceral input to the spinal cord compared to their somatic counterparts, the extent of the visceral afferent-mediated responses is noteworthy.

### Monoaminergic Modulation of Motor Output

The descending monoaminergic systems are known to strongly modulate somatic afferent and efferent activity (e.g. [22], [24]) and their activity level varies with behavioral state [81], [82]. However, few studies have looked at modulatory actions of these systems on visceral afferent dependent activity. In this study, all three monoamines tested potently depressed splanchnic-evoked excitatory actions on ventral root responses (VRP depression).

Whole-cell recordings assessed direct actions on SPNs. All monoamines preferentially facilitated SPN excitability. 5HT always depolarized SPNs and both 5HT and NA always increased SPN firing properties. Since NA and DA could also lead to membrane hyperpolarization and DA to reduced excitability in a minority of SPNs, it is clear that the monoamines can act bidirectionally. Differences in modulatory actions may be related to actions on differentiable subpopulations of SPNs including those previously differentiated by difference in cell membrane properties [54].

#### Observed monoamine modulatory actions compare favorably to previous studies in both rat and cat

5HT induced membrane depolarizations in the neonatal rat *in vitro* [47], [83], [84] and observed excitability increases were previously seen with iontophoretically applied 5HT in SPN firing in cats [85]. The mixed membrane responses observed here with NA are also consistent with that reported in the neonatal rat and adult cat [49], [51], [52], [86]. Similarly, mixed DA responses on membrane potential were reported previously in the neonatal rat [48], while opposite actions on SPN firing have been reported in the adult rat [87] and cat [88].

Given the generally increased excitability of sympathetic efferents (SPNs) produced by the monoamines but decreased sensory-evoked efferent responses, the monoamines appear to modulate SPN activity away from sensory control, thereby shifting the influence of sympathetic function toward centrally driven events. As we also showed that this trend occurs in a dose-dependent manner, the strength of descending monoaminergic modulatory drives presumably shape the physiological level of afferent influence, as explored further below.

### Dorsal Root Potentials and Primary Afferent Depolarization

Splanchnic and sympathetic chain stimulation produced a DRP, a measure of PAD, in dorsal roots of multiple thoracic spinal segments. The measured DRP duration was comparable to that reported previously after the stimulation of muscle and cutaneous afferents (e.g. [33], [67], [89]). These DRPs were also abolished by bicuculline as similarly observed in cutaneous and muscle afferent evoked DRPs, supporting PAD as mediated by last-order GABAergic interneurons ([32], [33]; but see [33]).

The average latency to DRP onset of visceral-afferent evoked responses occurred much later than those reported in response to low threshold segmental afferent stimulation [68], [78], here being greater than 30 ms after the first afferent volley was detected. Visceral afferent evoked DRPs have also been reported to occur much later than the shortest latency responses observed following cutaneous afferent stimulation [36]. As these DRPs also occurred ∼20 ms after VRPs were recorded in our preparation, distinct afferent populations and/or distinct last-order interneuronal populations are implicated in their occurrence. Multi-segmental longer-latency DRPs have been observed previously with dorsal root stimulation [65], and it is conceivable that the long-latency visceral afferent-evoked DRPs seen presently converges onto a common set of interneurons. In contrast, both cutaneous and muscle afferent-evoked PAD are well known to utilize much shorter latency pathways [68], [90]. Short-latency cutaneous and muscle afferent pathways may be required for greater temporal and spatial resolution as well as local negative feedback control mechanisms not as relevant to the control of visceral afferent signaling.

### Visceral Afferent-evoked Field Potentials

EFPs were recorded to investigate the modulation of visceral afferent synaptic transmission in the spinal cord. Tracking experiments identified maximal responses in the deep dorsal horn, consistent with the location of spinal neurons activated by visceral afferents in the cat and rat [12], [19] as well as anatomical labeling studies of visceral afferent projections in the spinal cord [10], [15]. While the earliest measured EFP onset was 4.5 ms, consistent with monosynaptic transmission [91], latencies were highly variable, averaging 13 ms. Such longer latencies may reflect di- or tri-synaptic actions. Alternatively, as components of the afferent volley could spread over many milliseconds (e.g. Figure 7) longer-latency EFPs may instead arise monosynaptically from afferents with slower conduction velocities. Further studies that use approaches to identify monosynaptic actions are warranted [68]. Regardless, given that the observed monoaminergic depressant actions occurred uniformly on evoked EFPs regardless of latency, observed actions can be conservatively interpreted as at least partly reflecting modulation of afferent monosynaptic transmission onto first-order interneurons in the deep dorsal horn.

### Modulation of Dorsal Root Potentials, Intraspinal Field Potentials and Dorsal Root Reflexes

The monoamines are known have differentiable depressant actions on somatic afferent interneuronal pathways [38], [39], but the modulatory actions of 5HT, NA, and DA on spinal visceral afferent processing is not well known. The reduction in population excitatory postsynaptic potentials (↓EFP) produced by the monoamines support a depression of visceral afferent input. This interpretation is consistent with previous work showing that the monoamines generally depress afferent-evoked monosynaptic transmission to individual neurons in the deep dorsal horn [37]. Depressed afferent transmission is consistent with the concomitantly observed reduction in reflex/VRP efferent responses.

Assuming observed EFPs reflect transmission at the first synapse in interneuronal pathways that generate the DRP (our measure of PAD), a reduction in the EFP would lead to a reduced DRP amplitude. This highlights the need for caution in interpreting reductions in the DRP as a reduction in presynaptic inhibition via depression of the interneuronal populations producing PAD. Here, the visceral-afferent evoked DRP was strongly depressed by the 5HT, NA, and DA, with IC_50_ values of 0.33, 0.27, and 3.9 µM, respectively. If the DRP reduction is an expression of PAD-evoked presynaptic inhibition, this effect suggests that descending monoaminergic systems strengthen visceral afferent evoked responses by reducing presynaptic inhibition. On the other hand, if the DRP reduction is due simply to reduced transmission in the stimulated afferents, then the conclusion is that descending monoamines serve to weaken afferent actions. In separate unpublished studies, evidence for depression of the DRP independent of afferent transmission was seen in response to muscle and cutaneous afferent stimulation [69], [92].

In the present study, the observed EFP results clearly support monoamine depressant modulatory actions at incoming afferent synapses. Evidence of additional distinct depressant actions on downstream interneurons that lead to the production of PAD is supported by two observations (Figure 8). First, the EFP recovered from depression after washout faster than did the DRP, implying the delayed DRP recovery was due to a temporally slower recovery in the PAD producing synapses. Second, at least for 5HT, the DRP was more strongly depressed than the EFP, suggesting additional modulatory actions on PAD producing synapses.

Overall then, it appears that the monoamines modify spinal processing of visceral afferent input in at least two ways: 1) decreasing visceral afferent transmission to the spinal cord (↓EFP) and 2) increasing repetitive synaptic actions by limiting PAD-mediated presynaptic inhibition (↓DRP). Differentiable sites of action with apparent opposite physiological consequence require more detailed studies to fully understand, but need not be dichotomous. For example, a reduction in primary afferent transmission (↓EFP) would reduce the influence of tonic low-frequency afferent activity on spinal circuits, while a reduced interneuronal activity-dependent presynaptic inhibition (↓DRP) would support a comparative facilitation of transmission during periods of higher frequency afferent activity.

Under control conditions evoked DRPs were usually of sufficient magnitude to recruit backward-propagating dorsal root reflexes. The monoamines 5HT, NA, and DA not only greatly depressed DRPs; they consequently also blocked the accompanying dorsal root reflexes. Dorsal root reflexes produce back-propagating spikes and can lead to peripherally-released neuromodulators (e.g. CGRP) and/or other signaling molecules that alter organ function and blood flow [93], [94]. The monoamines may therefore also play a role in reducing peripheral sensitization via a depression of dorsal root reflexes. Further study is needed to assess this intriguing control of sensory actions at their peripheral endings by descending modulatory systems.

### Putative Sites of Action

As there are many monoaminergic receptor subtypes present in spinal neurons and primary afferents (see [95] for review), the monoamines can affect neuronal function by activating metabotropic receptors (and ionotropic for 5HT_3_) that both modulate ion channels directly and/or modulate common signal transduction pathways (e.g. [96], [97]). Observed similarities and differences in action between the monoamines at a given site could thus be based on the complement of receptors expressed. For example, 5HT_1_, D_2-like_ and α_2_ receptors are all G_i_-coupled, and co-localization of these receptors at the same site may lead to comparable actions. Differing receptor expression patterns, actions on different signal transduction pathways, or dose-dependent differences in receptor affinity would be expected to lead to more complex actions. One such difference was observed with DA, where lower doses could facilitate the DRP while larger doses were depressant. Indeed, different DA receptors having different affinity has been shown in the pre-frontal cortex, where low doses of DA preferentially produce actions via D_1-like_ receptors and higher doses of DA masked these effects by activation of D_2-like_ receptors [98], [99].

**Figure 10:**
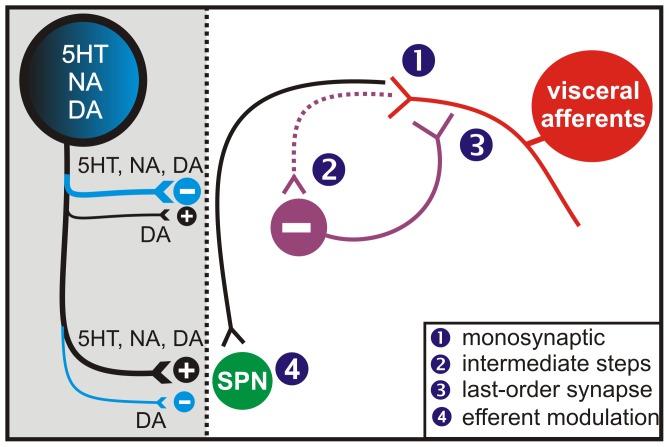
Potential sites of action of monoamines. 5-HT, NA and DA may exert actions on one or many of the following locations: 1) presynaptically directly inhibiting afferent transmission to the spinal cord, 2) inhibiting synaptic transmission to putative interneurons or altering their excitability so that fewer are recruited to produce PAD, or 3) inhibiting last order GABAergic transmission to the afferents producing PAD. 4) Monoamines also usually act to increase SPN excitability.

While a determination of receptor subtype identity and their specific sites of action is well beyond the scope of the present study, a schematic of these potential locations is conceptually instructive and shown in Figure 10. The site of modulation may therefore be: (1) presynaptically, by directly inhibiting afferent transmission to the spinal cord, (2) postsynaptically, inhibiting synaptic transmission or altering the excitability of interposed interneurons so that fewer are recruited to produce PAD, (3) inhibiting transmission of the last-order interneurons producing PAD, and/or (4) directly on SPNs to alter efferent excitability.

In summary, this work combined used of a novel *in vitro* spinal cord/sympathetic chain preparation with cellular studies of SPNs in a slice preparation to demonstrate that the monoamines all broadly act to facilitate sympathetic efferent activity while simultaneously suppressing visceral afferent evoked actions. These results lay the foundation for subsequent studies on central sites of action as well as studies on the behavioral relevance of such wide-ranging modulatory actions on the spinal autonomic nervous system.
